# Molecular mechanisms underlying the maintenance of terminal differentiation in Arabidopsis guard cells

**DOI:** 10.3389/fpls.2026.1952204

**Published:** 2026-09-15

**Authors:** Yongqiang Chen, Ruiying Li

**Affiliations:** 1 National and Local Joint Engineering Laboratory of Efficient and High-Quality Cultivation and Deep Processing Technology of Characteristic Fruit Trees in Southern Xinjiang, College of Horticulture and Forestry, Alar, China; 2 National Key Laboratory of Crop Genetic Improvement, Hubei Hongshan Laboratory, Huazhong Agricultural University, Wuhan, China; 3 Characteristic Forest and Fruit Technology Innovation Center of Southern Xinjiang in Xinjiang Production and Construction Corps (XPCC), Alar, China

**Keywords:** FAMA, guard cell, HAC1, PRC2, RBR1, SWI/SNF, terminal differentiation

## Abstract

In many plant species, guard cells retain their distinctive morphology and physiological function for extended periods after differentiation and maturation. How this terminally differentiated state is actively maintained over time is not fully understood, but recent studies have begun to reveal the underlying mechanisms. Elucidating the underlying mechanisms in *Arabidopsis* guard cells may provide valuable insights into the maintenance of differentiated identities in other plant cell types. In this review, we synthesize recent findings showing that guard cell identity is secured by two interconnected layers: PRC2-mediated H3K27me3 deposition silences stomatal stem cell genes, while sustained expression of FAMA, supported by SWI/SNF and HAC1-dependent chromatin remodeling, reinforces the mature transcriptional program. Permanent cell cycle exit may serve as an additional safeguard against dedifferentiation, as aberrant cell cycle reactivation triggers fate resetting. This framework highlights the combined action of two interconnected layers: repression of stomatal stem cell genes, coupled with sustained expression of the mature transcriptional program, with cell cycle exit providing additional protection. Such a framework may serve as a useful reference for studying terminal differentiation maintenance in guard cells and other post-mitotic cell types.

## 1 Introduction

Multicellular organisms are composed of a large number of highly specialized cell types, which are typically derived from pluripotent stem cells through differentiation (Thomson et al., 1998; Murry and Keller, 2008; Nichols and Smith, 2009). Once terminally differentiated, these cells rarely revert spontaneously to an undifferentiated state. Terminal differentiation represents the final stage of cellular specialization, characterized by permanent cell cycle withdrawal and the acquisition of highly specialized structural and functional properties (Buttitta and Edgar, 2007; Ma et al., 2019). Unlike stem cells or meristematic cells that retain proliferative capacity, terminally differentiated cells are the ultimate products of developmental trajectories, typically functioning for extended periods without division (Torii, 2015; Simmons and Bergmann, 2016; Han and Torii, 2019; Aughey, 2023). Although the transition from stem cells to differentiated cells has been extensively studied, how cells maintain their terminally differentiated identity over the long term, preventing lineage reversal or dedifferentiation, remains poorly understood.

Currently, substantial direct molecular evidence for terminally differentiated fate maintenance in plants comes from *Arabidopsis* stomatal guard cells (Lee et al., 2014; Matos et al., 2014; Yang et al., 2014; Lee et al., 2019; Liu et al., 2024). As highly specialized, terminally differentiated cells, guard cells (GCs) persist on the leaf surface for extended periods once morphogenesis and functional maturation are complete. To maintain their specialized function, the identity of GCs and the transcriptional state that underpins it must block stomatal lineage reversal or dedifferentiation, as well as suppress cell cycle progression. Moreover, maintaining stomatal function requires not only the long-term repression of non-stomatal lineage genes but also sustained transcription of genes essential for stomatal activity. What molecular machinery prevents guard cells from reverting to stem cell-like states? How is the stable expression of stomatal function genes achieved over time? In this review, we integrate the limited but key evidence accumulated from GCs to provide valuable insights for deciphering terminal-fate maintenance in other plant cell types.

## 2 Molecular mechanisms of terminal differentiation maintenance in guard cells

### 2.1 Transcription factors involved in guard cell differentiation and maturation

Stomatal development in *Arabidopsis* proceeds through a well-characterized sequence, from stomatal lineage precursor cells to guard mother cells (GMCs), which then execute a single symmetric division to generate a pair of guard cells. Upon completing morphogenesis and acquiring functional maturation, these guard cells become terminally differentiated and must maintain their specialized identity for extended periods (Torii, 2021). The bHLH transcription factor FAMA acts as a master regulator of guard cell differentiation. In *fama* loss-of-function mutants, GMCs fail to transition into GCs and instead undergo repeated longest-wall divisions, a distinctive type of equal division, to generate “caterpillar”-like cell clusters. Conversely, overexpression of *FAMA* leads to the production of numerous single GCs in the epidermis and mesophyll layers, and these cells already exhibit characteristics of mature GC identity (Ohashi-Ito and Bergmann, 2006). These results indicate that FAMA is sufficient to drive terminal differentiation from GMC to GC. In addition, the R2R3-MYB family members FOUR LIPS (FLP) and MYB DOMAIN PROTEIN 88 (MYB88) also play important roles in the GMC-to-GC transition. The *flp myb88* double mutant exhibits phenotypic similarities to *fama*, and the FLP and MYB88 share overlapping functions in promoting proper stomatal differentiation (Lai et al., 2005; Xie et al., 2010; Lee et al., 2014).

Recently, the downstream regulatory network of FAMA has been further elucidated. FAMA directly induces the expression of two transcription factor genes, *WASABI MAKER* (*WSB*) and *STOMATAL CARPENTER 1* (*SCAP1*) (Shirakawa et al., 2025). In the stomatal lineage, *WSB* expression occurs earlier than *SCAP1* and has a narrow temporal window, whereas *SCAP1* expression rises at later stages and persists in mature GCs (Negi et al., 2013; Castorina et al., 2016). WSB and SCAP1 cooperatively promote guard cell differentiation, as *wsb scap1* double mutant exhibits severe defects in stomatal maturation, failing to produce morphologically normal, functional guard cell pairs with properly formed pores. Instead, stomatal lineages frequently arrest at immature stages or develop highly aberrant structures, including poreless guard cells, multi-cell stomatal units, clustered guard cell pairs, and disorganized stomatal architecture (Shirakawa et al., 2025). Moreover, the *scap1* loss-of-function mutants exhibit irregular GC morphology with floppy ventral inner walls, and the plants fail to respond properly to environmental changes in stomatal movement (Negi et al., 2013). Transcriptomic data indicate that SCAP1 ensures the complete morphological and functional maturation of GCs by coordinating the expression of stomatal movement-related genes and cell wall synthesis-related genes (Negi et al., 2013).

Taken together, GC differentiation and maturation are coordinately controlled by FAMA as well as FLP/MYB88. The FAMA-SCAP1 regulatory axis ensures that guard cells acquire full stomatal movement functions. FLP and MYB88, in addition to restricting GMCs to a single symmetric division, also contribute to promote differentiation. The maturation process driven by these transcription factors lays the molecular foundation for the long-term maintenance of the terminally differentiated state of GCs. The roles of these transcription factors in mature guard cells, and the mechanisms that sustain their expression, are discussed in the following sections.

### 2.2 Epigenetic locking: polycomb-mediated silencing

Terminally differentiated GCs must maintain their identity over the long term and prevent reversion to a stem cell-like state. In recent years, accumulating evidence has highlighted that Polycomb-Group (PcG)-dependent deposition of the repressive histone mark H3K27me3 is critical for maintaining the terminally differentiated state of guard cells (Lee et al., 2014, 2019).

Early epigenetic clues come from studies of introduction of FAMA-GFP transgene (*FAMA^trans^*) under its own promoter to wild-type *Arabidopsis*, the mature GCs of *FAMA^trans^* lines underwent fate resetting: they initiated asymmetric divisions and re-activated stomatal development programs within the original guard cells, ultimately forming an aberrant structure termed “stoma-in-stoma” (SIS) (Lee et al., 2014). Within SIS guard cells, early stomatal lineage marker genes (*SPCH*, *TMM* and *MUTE*) were reactivated, whereas late differentiation marker gene *KAT1* were lost (Lee et al., 2014). Further analysis revealed that the repressive histone marker H3K27me3 at the *SPCH* and *MUTE* loci were significantly reduced in GCs exhibiting the SIS phenotype, suggesting that the disruption of epigenetic silencing is a direct cause of fate resetting (**Figure 1**). Consistent with this, overexpression of *CURLY LEAF* (*CLF*), a core component of the PRC2 complex, alleviated the SIS phenotype (Lee et al., 2014). More directly, specific knockdown of *CLF* and its homolog *SWINGER* (*SWN*) driving by *FAMA* promoter reduced H3K27me3 levels in GCs and resulted in a low frequency of fate resetting (**Figure 1**) (Lee et al., 2019). These results demonstrate that PRC2 complex-mediated H3K27me3 modification is essential for maintaining the terminally differentiated state of GCs.

**Figure 1:**
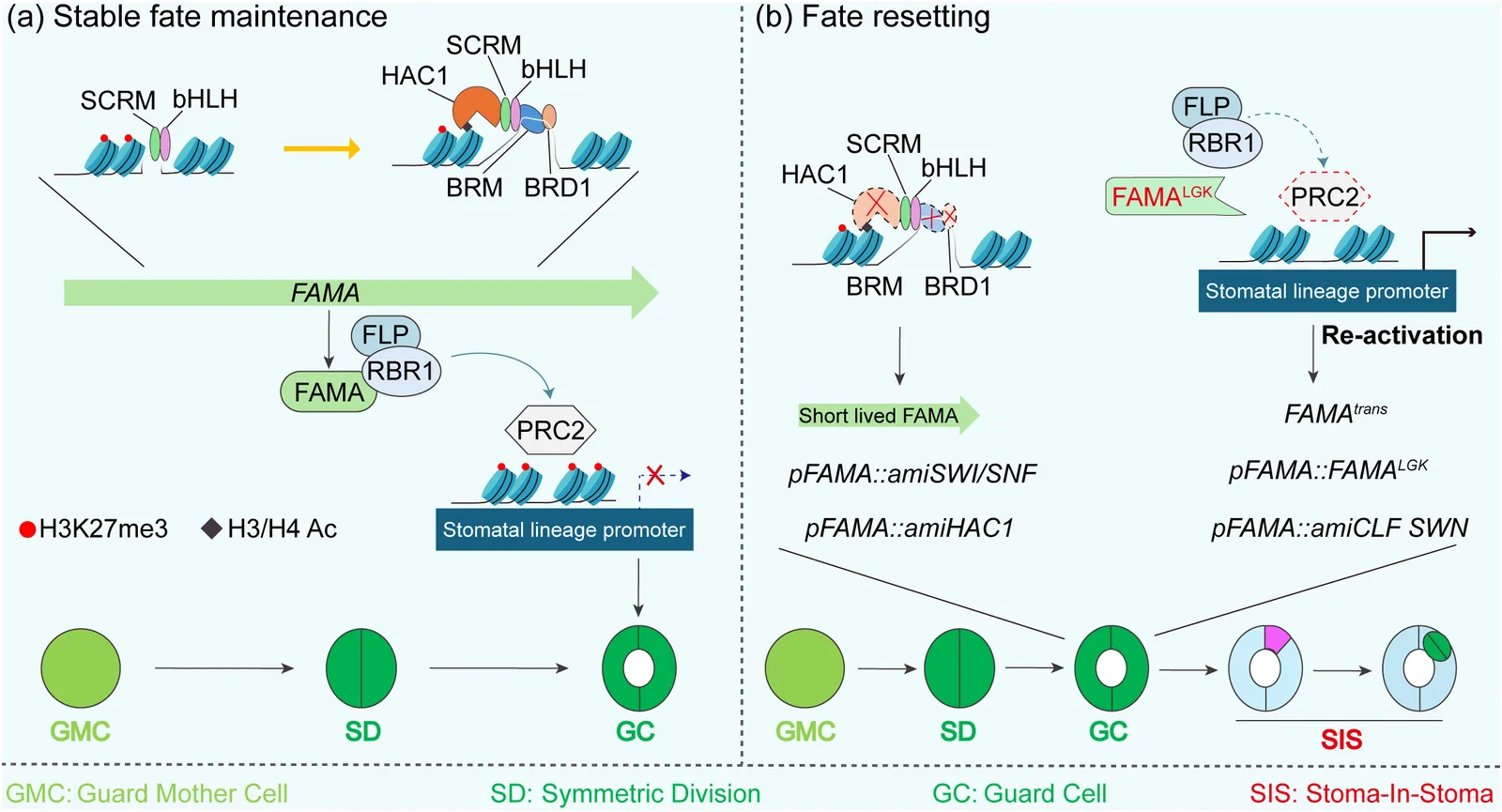
A model for terminally differentiated fate maintenance in *Arabidopsis* guard cells. **(a)** The terminal bHLH transcription factor FAMA drives guard cell differentiation. Following differentiation, FAMA recruits the retinoblastoma-related protein RBR1 via its LxCxE motif, forming the FAMA-RBR1 complex. The complex deposits the repressive histone mark H3K27me3 at early stomatal lineage genes loci (*SPCH*, *MUTE*), through recruitment of the PRC2 complex (containing CLF and SWN), thereby ensuring long-term transcriptional silencing. Sustained expression of FAMA in mature guard cells depends on the chromatin remodeling SWI/SNF complex and the histone acetyltransferase HAC1, which maintain an open chromatin configuration at the *FAMA* locus. Thus, FAMA, RBR1, PRC2, SWI/SNF, and HAC1 collectively constitute a regulatory network that locks in the terminally differentiated state through epigenetic silencing and chromatin remodeling. **(b)** However, disruption of the FAMA-RBR1 interaction (as in the *FAMA^LGK^*), knockdown of RBR1, or depletion of PRC2 components compromises H3K27me3 deposition and releases the repression of early stomatal lineage genes such as *SPCH* and *MUTE*. Consequently, mature guard cells re-enter the cell cycle and undergo asymmetric divisions, reactivating the entire stomatal developmental program within the original guard cells and ultimately forming the stoma-in-stoma (SIS) phenotype. Notably, ectopic expression of the G2/M cyclin CYCA2;3 in mature guard cells similarly triggers asymmetric divisions and fate reversal, indicating that aberrant cell cycle activation is sufficient to disrupt the terminally differentiated state. Known factors or mutants that alter the terminally differentiated state of guard cells: *pFAMA*::*amiSWI/SNF*, *pFAMA*:: *amiHAC1*, *FAMA^trans^*, *FLP^trans^*, *pFAMA*::*FAMA^LGK^*, *pFAMA*:: *ami CLF SWN*, *pFAMA*::*CYCA2.3*.

Notably, the *FLP^trans^* also induced the SIS phenotype, suggesting that FAMA and FLP may share maintenance mechanisms (Lee et al., 2014). Both FAMA and FLP are known to interact with the RETINOBLASTOMA-RELATED PROTEIN 1 (RBR1). Specific downregulation of *RBR1* expression under the *FAMA* promoter (*pFAMA:amiR-RBR1*) similarly caused fate resetting in mature GCs (Lee et al., 2014). Importantly, the FAMA protein contains a canonical RBR1-binding motif (LxCxE). Mutation of this motif to LGK (*pFAMA: FAMA^LGK^-YFP*) recapitulated the SIS phenotype (**Figure 1**) (Matos et al., 2014). This provides direct evidence that the physical integrity of the FAMA-RBR1 complex is required for the maintenance of guard cell terminal differentiation.

Collectively, the current model proposes that in normally differentiating guard cells, FAMA and FLP both interact with RBR1 (Lee et al., 2014), which recruits the PRC2 complex to specifically enrich at the promoter regions of key stomatal lineage genes (*SPCH*, *MUTE*). PRC2 further catalyzes the deposition of the repressive histone modification H3K27me3, thereby establishing a locally transcriptionally repressive chromatin microenvironment. This process permanently silences early stomatal lineage genes, prevents the re-entry of cells into proliferative and differentiation programs, and locks GCs in a stable terminally differentiated state (**Figure 1**).

### 2.3 Chromatin remodeling during terminal differentiation

To sustain the terminally differentiated state, guard cells must preserve FAMA-dependent transcriptional programs across their functional lifespan. Direct evidence comes from a “short-lived FAMA” reporter line (*FAMApro::gFAMA-YFP/fama-1*), in which gFAMA-YFP is expressed for a shorter period in each developing guard cell (McKown et al., 2023; Liu et al., 2024); in this line, FAMA expression is prematurely terminated before guard cells fully mature. These guard cells fail to establish proper identity, indicating that sustained FAMA expression is required for terminal differentiation (McKown et al., 2023; Liu et al., 2024). This requires the maintenance of an active chromatin configuration at the *FAMA* locus. The bHLH transcription factors (SPCH, MUTE, FAMA and SCRMs) in the stomatal lineage typically function as heterodimers and can access targets located in repressive chromatin regions (Kim and Torii, 2024; Liu et al., 2024; Chen et al., 2025; Chen and Zhang, 2026). Based on the current model, FAMA-SCRM recruits the ATPase-dependent chromatin remodeling SWI/SNF complex and the histone acetyltransferase HAC1, which are proposed to evict nucleosomes and acetylate histone tails, thereby inducing local chromatin reprogramming and sustaining expression of genes required for stomatal cell fate determination at different stages (Liu et al., 2024). *FAMA* promoter-directed amiRNAs targeting SWI/SNF subunits or HAC1 induce successive ectopic asymmetric divisions in GCs, which occasionally forming GCs or tumors inside of GCs. In these amiRNA lines, early stomatal lineage markers such as SPCH and MUTE were expressed in GCs, whereas the features of fully mature guard cells failed to be established (**Figure 1**) (Liu et al., 2024). Thus, reducing SWI/SNF or HAC1 activity blocks the transition to terminally differentiated guard cells.

Interestingly, the phenotypes of these amiRNA lines closely resemble that of a “short-lived FAMA” reporter line (**Figure 1**) (Liu et al., 2024). This observation indicates that the late-stage activity of FAMA is not autonomously maintained but depends on the sustained function of HAC1 and the SWI/SNF complex. In other words, the function of “late FAMA” critically relies on chromatin remodeling factors, which govern not only its expression but also its functional competence to drive and accomplish guard-cell terminal differentiation.

In animal terminally differentiated cells, differentiation transcription factors similarly recruit SWI/SNF complexes and histone acetyltransferases (HATs) to open chromatin and activate downstream genes. Chromatin modifying complexes are involved in maintaining overall neuronal lineage fate (Fryer and Archer, 1998; Yudkovsky et al., 1999; Ho and Crabtree, 2010). For instance, loss of *SWI/SNF* in *Drosophila* neural stem cells cause transit-amplifying progeny to revert to stem cell-like fates, leading to neoplastic growth and tumor formation (Eroglu et al., 2014). For guard cells, chromatin remodeling during differentiation is an essential step for acquiring mature identity, but whether and how these open regions are maintained after differentiation remains to be investigated.

### 2.4 Cell cycle exit is required for terminal differentiation

Terminally differentiated guard cells must maintain long-term suppression of cell cycle activity to prevent loss of identity. In animals, the retinoblastoma (RB1) protein is a tumor suppressor that negatively regulates cell cycle progression through its interaction with E2F/DP transcription factors (Singh et al., 2016; Desvoyes and Gutierrez, 2020). In plants, the retinoblastoma-related protein RBR1 plays a crucial role in maintaining guard cell terminal differentiation. Loss of RBR1-mediated repression of SPCH, a master regulator of asymmetric division in the stomatal lineage, leads to ectopic divisions and likely contributes to fate resetting (Weimer et al., 2012; Lee et al., 2014; Matos et al., 2014). Importantly, it has been shown that RBR1 targets E2F-regulated genes, including *CDKB1* and *CDC6* (Nowack et al., 2012; Weimer et al., 2012), suggesting that RBR1 may also promote terminal differentiation maintenance by repressing key cell cycle factors. On the other hand, according to the current working model, RBR1 is proposed to recruit the PRC2 complex to deposit H3K27me3 at loci such as *SPCH*, establishing long-term epigenetic silencing (Simmons and Bergmann, 2016). Thus, RBR1 serves as a key hub connecting transcriptional repression, cell cycle control, and epigenetic locking to maintain GC terminal differentiation, demonstrating that the block to proliferation is actively maintained rather than being a passive consequence of differentiation.

The importance of cell cycle suppression is further supported by a gain-of-function experiment. Ectopic expression of the cyclin *CYCA2;3* driven by the *FAMA* promoter (*pFAMA: CYCA2;3*) in mature guard cells caused asymmetric divisions in a subset of GCs, and the daughter cells re-expressed stomatal lineage stem cell genes, while mature guard cell identity markers were lost (Yang et al., 2014). Interestingly, ectopic expression of *CYCDs* triggers re-entry of the cell cycle in fully differentiated GCs without fate reversion. Notably, these divisions represent symmetric divisions, yet they do not occur along the longest cell wall (Yang et al., 2014; Han et al., 2018; Weimer et al., 2018). Thus, cell-cycle re-entry is a necessary but not sufficient condition for cell-fate reversal. While cell-cycle re-entry acts as an obligate prerequisite, yet additional regulatory inputs are required to trigger full loss of guard-cell identity.

In many plant species, cell cycle exit is correlated with the onset of endoreplication, a process during which cells duplicate their nuclear DNA in the absence of cell division (Polyn et al., 2015). Interestingly, *Arabidopsis* trichomes achieve functional specialization through endoreduplication. Nevertheless, like guard cells, trichomes also require permanent cell cycle withdrawal, which is the hallmark of terminal differentiation (Buttitta and Edgar, 2007; Ma et al., 2019). Researchers found that impairment of endoreplication causes newly formed trichome cells to dedifferentiate. Dedifferentiated trichomes undergo repeated cell divisions and become reintegrated into the epidermal cell layer, acquiring the characteristic features of surrounding pavement cells (Hülskamp, 2004; Bramsiepe et al., 2010). These observations raise two important points. First, permanent cell cycle withdrawal, rather than the absence or presence of endoreplication per se, is the defining feature of terminal differentiation shared by guard cells and trichomes alike. Second, while the molecular strategies for achieving functional specialization differ between these cell types, the requirement for an active block to proliferation appears to be a common principle. Together, these findings reinforce the view that terminally differentiated cells, regardless of whether they employ endoreplication, must actively safeguard their post-mitotic status to preserve cell identity.

## 3 Discussion

It is now increasingly evident that preserving differentiated cell states requires an active, continuous process throughout a cell’s lifetime to prevent identity loss or aberrant reprogramming. Using *Arabidopsis* guard cells as a model, studies over the past decade have begun to unravel the molecular mechanisms that maintain terminally differentiated cell identity (Torii, 2015; Simmons and Bergmann, 2016; Han and Torii, 2019).

Based on the evidence summarized in the preceding sections, the maintenance of guard cell terminal differentiation emerges from the coordinated action of multiple regulatory layers. First, the terminal transcription factor FAMA, together with FLP/MYB88, drives guard cell differentiation and maturation. Following differentiation, the FAMA-RBR1 interaction recruits PRC2 to deposit H3K27me3 at stomatal stem cell regulators such as *SPCH* and *MUTE*, establishing long-term transcriptional silencing that locks in the differentiated state. In a separate layer, sustained expression of FAMA in mature guard cells depends on SWI/SNF and HAC1-mediated chromatin remodeling, which maintains an open chromatin configuration at the *FAMA* locus and reinforces the mature transcriptional program (**Figure 1**). Finally, RBR1 also connects this epigenetic locking to permanent cell cycle exit, actively preventing dedifferentiation. Taken together, the maintenance of terminally differentiated GC identity can be viewed as involving two interconnected layers: repression of stomatal stem cell regulators, coupled with sustained expression of key transcription factors such as FAMA. While this framework is supported by current evidence, the relative contributions of these layers and their mechanistic interplay remain important areas for further investigation.

Despite these advances, several open questions remain. First, stable FAMA expression is central to GC fate maintenance. FAMA-SCRM cooperates with SWI/SNF and HAC1 to maintain open chromatin at the FAMA locus and sustain its own expression (Liu et al., 2024), yet the stability and regulation of this positive-feedback loop remain unclear. How mature guard cells reconcile these opposing chromatin activities, SWI/SNF-dependent chromatin opening and PRC2-dependent compaction, to simultaneously maintain fate-gene expression and repress stem-cell program is an outstanding question. Second, although cell-cycle exit is required for fate maintenance, the contribution of endoreplication remains under-explored. Unlike GCs, many plant cell types (trichomes, pavement cells) undergo endoreplication during differentiation. Impaired endoreplication in trichomes can drive dedifferentiation, supporting a role for endoreplication in locking terminal identity (Bramsiepe et al., 2010). Further genetic manipulation in trichomes, pavement cells and root cells is needed to validate this hypothesis.
